# *CHD7* gene polymorphisms in female patients with idiopathic scoliosis

**DOI:** 10.1186/s12891-019-3031-0

**Published:** 2020-01-10

**Authors:** Karolina Borysiak, Piotr Janusz, Mirosław Andrusiewicz, Małgorzata Chmielewska, Mateusz Kozinoga, Tomasz Kotwicki, Małgorzata Kotwicka

**Affiliations:** 1 Department of Cell Biology, Poznan University of Medical Sciences, Poznań, Poland; 2 Department of Spine Disorders and Pediatric Orthopedics, Poznan University of Medical Sciences, Poznań, Poland

**Keywords:** Idiopathic scoliosis, IS; chromodomain helicase DNA binding protein 7, CHD7; single nucleotide polymorphisms, SNP; cobb angle

## Abstract

**Background:**

The *CHD7* (chromosome domain helicase DNA binding protein 7) gene has been associated with familial idiopathic scoliosis (IS) in families of European descent. The *CHD7* single-nucleotide polymorphisms have never been studied in Polish Caucasian IS patients.

**Methods:**

The aim of this study was to investigate the relationship of *CHD7* gene polymorphisms with susceptibility to or progression of IS in Polish Caucasian females. The study group comprised 211 females who underwent clinical, radiological and genetic examination. The study group was analyzed in three subgroups according to: (1) Cobb angle (Cobb angle ≤30° vs. Cobb angle **≥**35°), (2) age of diagnosis (adolescent IS vs. early-onset IS) and (3) rate of progression (non-progressive vs. slowly progressive vs. rapidly progressive IS). The control group comprised 83 females with no scoliosis and with a negative family history who underwent clinical and genetic examination. In total six *CHD7* gene polymorphisms were examined. Three polymorphisms (rs1017861, rs13248429, and rs4738813) were examined by RFLP (restriction fragment length polymorphism) analysis, and three were quantified by Sanger sequencing (rs78874766, rs4738824, and rs74797613).

**Results:**

In rs13248429, rs78874766, and rs74797613 polymorphisms only the wild allele was present. The rs1017861 polymorphism demonstrated an association with IS susceptibility (*p* < 0.01). Two polymorphisms, rs1017861 and rs4738813, were associated with curve severity and progression rate (*p* < 0.05). None of the evaluated polymorphisms in *CHD7* gene showed any association with the age of IS onset.

**Conclusions:**

The polymorphism rs1017861 in *CHD7* gene showed an association with IS susceptibility. Two polymorphisms (rs1017861 and rs4738813) were associated with curve severity and progression rate. None of the evaluated polymorphisms in *CHD7* gene showed any association with the age of IS onset. Further evaluation of *CHD7* gene should be considered as IS modifying factor.

## Background

Idiopathic scoliosis (IS) is defined as three-dimensional structural spine deformation, with lateral curvature greater than 10 degrees measured on standing radiograph according to the Cobb method, with vertebral rotation. The prevalence of IS is estimated from 1 to 3% in the adolescent population [1]. The etiology of IS remains unknown and is described as a multifactorial disease with strong genetic influence [2].

The inheritance of scoliosis in five generations was described by Garland as early as 1934 [2]. It is indicated that the incidence of scoliosis in first-degree relatives is significantly higher than in the general population. In addition, SI concordance (meaning that both twins have this pathology) in monozygotic twins is higher in comparison to dizygotic twins [3], which further indicates the influence of genetic factors [3–6]. Thus, it justifies studies undertaken to estimate the relationship of genetic factors with scoliosis susceptibility. However, a panel of genes that could be useful in scoliosis diagnostic and treatment planning has not been defined yet.

Association between IS occurrence or progression and single nucleotide polymorphisms (SNPs) were reported in several genes [7, 8]. Among them, the gene encoding the chromodomain helicase DNA binding protein 7 (*CHD7*) was highlighted [9].

The *CHD7* gene (OMIM# 608892) is located on chromosome 8 (8q12.2), 188kbp in length and composed of 42 exons. The CHD7 protein is a member of the chromodomain helicase DNA binding domain family of ATP-dependent chromatin remodeling enzymes. These proteins are found in nucleolus and nucleoplasm [10, 11]. Mutations arising de novo in this gene are the major cause of the CHARGE syndrome, a genetic disease characterized by a coexistence of congenital deformation including coloboma of the eye, heart defects, choanal atresia, severe Retardation of growth and development, genital and ear abnormalities. CHARGE syndrome is seen in 1:16,000 births worldwide [12] and, importantly, 60% of those patients develop a scoliosis phenotype [13].

Although the *CHD7* was the first described gene linked to idiopathic scoliosis, there are only two studies concerning polymorphisms of this gene in IS [9, 14]. Gao et al. reported that *CHD7* gene polymorphisms were related to familial idiopathic scoliosis (FIS) in 52 families of European descent [9]. However, Tilley et al. in the evaluation of 22 genotyped SNPs in the *CHD7* gene in 244 FIS families, found no association between SNPs and the FIS. What is more, a meta-analysis of the two study samples indicated no association of the *CHD7* gene with the phenotype of FIS [14].

Currently, an objective and unambiguous assessment of the individual risk of scoliosis progression at the time of diagnosis is not available. Evaluation of this indicator would be very valuable for determining the optimal treatment plan. Thus, IS can be described as a multifactorial disease, including contributing effects of both genes and environmental factors [15].

Taking into consideration insufficient data concerning the association of IS and *CHD7*, this study aimed to evaluate *CHD7* polymorphisms in Caucasian females with IS in term of IS susceptibility, age of IS onset, curve severity and progression rate.

## Methods

### Study subjects

Two hundred eleven Caucasian females with IS were recruited as cases in one Central European country (Poland) from March 2010 until June 2014. All of them underwent clinical, radiological and genetic examination. The inclusion criteria were: (1) clinically and radiologically confirmed IS diagnosis, (2) no coexisting orthopedical or neurological disorders and (3) achieved skeletal maturity. Skeletal maturity was defined as follows: (1) the age of at least 16 years at the evaluation, (2) more than 2 years after menarche, (3) Risser sign of 4 or 5, and (4) the end of the growth process, defined as the height increase of less than 1 cm during the previous 6 months. The Cobb angle was assessed at the final follow-up at the skeletal maturity. The age of 16 was consider as one of the maturity indicators and its evaluation was one of the inclusion criterion for patienits with slowly progressive idiopathic scoliosis and nonprogressive idiopathic scoliosis. In surgically treated patients, the progressive form of IS was documented radiologically, so the maturity criterium was not needed.

In each case, the age of onset of IS, the age of menarche and treatment history were noted. The radiological examination was performed using standing posteroanterior X-rays. The curve pattern, Cobb angle and Risser sign were measured by an experienced spine surgeon. The X-rays evaluation was performed at beginning of treatment, follow-up visits and the final follow-up at the end of the treatment or the pre-operative visits. To calculate the progression rate, the Cobb angle values obtained during follow-up visits were compared and presented as a degrees of the curve progression per month. Progression rate was defined as the change of Cobb angle value on the two consecutive X-rays which were taken at 12-month time intervals, expressed in degrees per month.

83 healthy females without IS were included in the control group. The inclusion criteria for the control group were: (1) meeting all the above-mentioned maturity indicators, (2) an angle of trunk rotation of less than 4° at examination with scoliometer (Adams’ forward bending test), and (3) a negative family history of idiopathic scoliosis. The radiological examination was not performed in the control group.

#### Patient subgrouping

Within the study group, clinically similar subgroups of patients were distinguished to establish the relation of polymorphisms with clinical form of IS. The first division included two subgroups depending on the curvature indicated by the Cobb angle at the end of treatment (Cobb angle above 35° and more versus Cobb from 10° to 30°). The second division included three subgroups depending on the progression rate of the Cobb angle curvature: (1) rapidly progressive idiopathic scoliosis (RP-IS), (2) slowly progressive idiopathic scoliosis (SP-IS) and (3) nonprogressive idiopathic scoliosis (NP-IS) [16]. The third division included two subgroups according to the age at the time of diagnosis: (1) early-onset idiopathic scoliosis (EOIS) – diagnosed at age 3–10 years and (2) adolescent idiopathic scoliosis (AIS) diagnosed at age ≥ 10 years.

### Genotype analysis

Genomic DNA was isolated from peripheral venous blood (collected in EDTA-containing disposable tubes; Sarstedt, Germany) with the use of AxyPrep Blood Genomic DNA Miniprep Kit (accordingly manufacturer protocol; Axygen, USA). Using NanoDrop ND-1000 spectrophotometer (Thermo Scientific, USA) the DNA yield and purity were assessed (A = 260 and A = 260/280 nm respectively) and the quality was analyzed by standard 0.8% agarose gel electrophoretic DNA separation in the presence of ethidium bromide.

#### DNA amplification

Specific DNA fragments were amplified using polymerase chain reaction (PCR) in a total volume of 20 μL. The reaction mixture included at final concentration: 1x KapaHiFi ready to use polymerase mix (KapaBiosystems; USA), 50–200 ng of gDNA, 100 nM of each sense and antisense primer (Genomed S.A., Poland). Primers’ characteristic and analysis method were given in Table 1. Primer3 software (http://bioinfo.ut.ee/primer3-0.4.0/) was used to design primers. The thermal profile included: initial denaturation (95 °C, 5 min), followed by thirty cycles of denaturation (95 °C–20 s), annealing (see Table 1 for annealing temperatures) and extension (72 °C, 20 s). Finally, one step of the elongation (72 °C, 3 min) followed by cooling to 12 °C were applied. 5 μL of the products were visualized in 1% agarose gel comparing to Nova 100 mass marker (Novazym, Poland).

**Table 1:** Characteristics of polymorphisms by measurement methods

SNP	Location	Nucleotide position	Analysis method	Enzyme	Nucleotide variants	Primer’s sequence 5′➔3′	Annealing temperature
rs78874766	Intronic	60,777,684	Sequencing	n/a	A/G	CATCGGGTCTCCTTTGGTTA	58 °C
rs4738824		60,777,762			A/G	ATCAATTCCCACTGCCTGAC	
rs74797613		60,777,857			A/G		
rs13248429	Exon 2	60,742,360	RFLP	*Nco*I	A/T	CAGCAGATGGGCAGCTATATG	
						ATTAGTCCTTGACCACTGTTTGATG	
rs1017861	Intronic	60,706,154		*Eco*RI	A/G	GTCCTGAGCTATGCAGTTCCA	61 °C
						ACCCCTTGTTCTTTCATCACCT	
rs4738813	Intronic	60,683,112		*Dpn*II	C/T	GCAAGCCTTCCATGGTTAAA	65 °C
						AGCTATGGACCTCTGCCATC	

*Abbreviations*: *SNP* single nucleotide polymorphism, *RFLP* restriction fragment length polymorphism, *n/a* not applicable

#### Polymorphisms evaluation

Three SNPs (rs13248429, rs1017861, rs4738813) were subjected to restriction fragment length polymorphism analysis (RFLP), and three additional SNPs were measured using sequencing (rs78874766, rs4738824, rs74797613). The methods were chosen based on the location of the polymorphisms studied and the possibility of selecting the appropriate restriction enzymes. Enzymes were selected using the NEBcutter V2.0 algorithm (New England Biolabs Inc.: http://tools.neb.com/NEBcutter2/).

RFLP reactions were performed accordingly to the enzyme manufacturer’s protocol (Thermo Scientific, USA) using 4 μL of the PCR product in 20 μL reaction volume and 5 μL of them were separated in 2% agarose gel in the presence of Nova 100 marker (Novazym, Poland). This enabled the homozygotic and heterozygotic alleles occurrence to be observed by gel analysis.

In the case of sequencing analysis of three polymorphisms, one 600 bp in length amplicon was used. The reaction products were purified accordingly to the manufacturer’s protocol (AxyPrep PCR Clean-up Kit; Axygen, USA) followed by sequencing (Genomed, Poland). 10% of the samples after the restriction reaction were also sequenced. All reactions (restriction enzyme analysis as well as sequencing reactions) were performed in duplicate.

### Statistical analyses

For the clinical and radiological studies, the minimum, maximum, mean and standard deviations were determined for age, the *age* of *first menstruation*, the age of scoliosis determination, Cobb angle, and the progression of curvature. These measures were compared between the subgroups with t-test or ANOVA, Mann-Whitney test or Kruskal-Wallis test depending on the statistical distribution assumptions. The Bonferroni correction was applied in case of multiple testing.

The distribution of alleles and genotypes were checked for Hardy-Weinberg equilibrium. The distribution of alleles and genotypes were compared between groups and subgroups described earlier with a Chi-square test. The probability of finding the odds ratio for the results of the above study was estimated, assuming a significance level of α = 0.05 and a desired 80% power. Also, the test power was evaluated for results that exceeded the significance level α = 0.05. Calculations were made using MedCalc 12.7.8.0. and G* Power 3.1.7. The D’ and LOD score (logarithm (base 10) of odds), were generated with Haploview software [17].

### Accordance with ethical standards

The Institutional Review Board at the Poznan University of Medical Sciences, Poznan, Poland, approved this study (Resolution No. 87/09). For each participant, written consent was obtained.

## Results

### Case-control study

The study group consisted of 211 females with IS (mean age of 16.8 ± 4.2 years old, range = 12.3–50.1). The mean Cobb angle was 39.6 ± 18.1 (10–114)° with a progression rate of 0.7 ± 0.9 (0–4) °/month. In the study group, the percentage of surgically treated cases was 26.5%. The control group consisted of 83 healthy females meeting inclusion criteria (mean age of 34.3 ± 6.7 years old, range = 16–55).

The genotypic frequencies of the SNPs followed the Hardy-Weinberg equilibrium. In three out of six of the SNPs (rs78874766, rs74797613, rs13248429) only the major allele was present either in patients or in controls. For this reason, we excluded these alleles from further evaluation. For statistical analyses, rs4738824, rs1017861, rs4738813 were assessed. The presented SNPs were not in linkage disequilibrium, polymorphisms are inherited separately, and strong evidence of recombination exist (Additional file 1). For the case-to-case study, among the subgroups divided by the Cobb’s angle curvature, progression rate and age of scoliosis appearance, there was no deviation from the Hardy-Weinberg equilibrium in genotypes frequencies.

In the case-control study, we observed significant differences in the rs1017861 polymorphism in alleles distribution (*P* = 0.0001) and in genotypes distribution for an independent and recessive model (*P* = 0.0006 and *P* = 0.002, respectively) (Table 2).

**Table 2:** Allele and genotype frequency (%) in patients with IS and healthy controls

Allele/genotype	Patients *N* = 211	Controls *N* = 83	Independent model *P*-value	P-value	OR (95% CI)
*rs1017861*					
G	87.7	74.6		**0.0001**^a^	2.4 (1.5–3.8)
A	12.3	25.4			
GG	77.2	55.4	**0.0006**	0.06^b^	3.3 (0.9–12.7)
GA	28	38.6			
AA	1.9	6.0		**0.002**^c^	0.4 (0.2–0.6)
*rs4738824*					
G	81.8	79.5		0.53^a^	0.84 (0.6–1.2)
A	18.3	20.5			
GG	65.9	63.9	0.54	0.47^b^	2.1 (0.6–7.9)
GA	31.7	32.1			
AA	2.4	4.8		0.84 ^c^	0.9 (0.5–1.6)
*rs4738813*					
T	68.7	69.3		0.97^a^	0.97 (0.66–1.44)
C	31.2	30.7			
TT	48.2	49.4	0.98	0.99^b^	0.99 (0.44–2.25)
CT	41	39.8			
CC	10.8	10.8		0.97^c^	0.96 (0.58–1.59)

*Abbreviations*: *IS* idiopathic scoliosis, *OR* odds ratio, *CI* 95% confidence interval, *P*-value for: ^a^ – alleles, ^b^ – dominant model, ^c^ – recessive model

significant *P*-values (<0.05) are in bold

For the rs4738824 and rs4738813 SNPs, no association of allele frequency with susceptibility to IS was found. Similarly, the genotype distributions showed no significant differences between patients with IS and healthy controls regardless of evaluated inheritance model (Table 2).

Our sample size for the case-control study was sufficient to detect differences in allele distribution for a *P*-value = 0.05 and statistical power of 80% as follows (presented as odds ratio (OR): for rs1017861 = 1.79, for rs4738824 = 1.69, and rs4738813 = 1.72.

### Case-only study

#### IS severity study and CHD7 polymorphisms

The characteristics of patients with IS, divided into two subgroups depending on the final Cobb’s angle curvature, is listed in Table 3. There were no statistical differences in age or first menarche onset (*p* ≥ 0.05), but the IS subgroups differed in maximal Cobb angle and progression rate (*P* = 0.0001). None of the patients with mild IS underwent surgical treatment.

**Table 3:** Characteristics of patients with different severity of IS

	Patients with Cobb angle ≤ 30°	Patients with Cobb angle ≥ 35°	*P*-value
Age (y)	16.3 ± 1.5 (15.0–23.3)	16.4 ± 5.0 (12.3–50.1)	0.88^a^
Age of menarche	12.9 ± 1.3 (10–16)	13.0 ± 1.2 (10–15)	0.64^b^
Cobb angle (°)	24.1 ± 5.4 (10–30)	52.4 ± 16.9 (35–114)	**0.0001**^**a**^
Progression rate (°/month)	0.15 ± 0.21 (0–0.99)	1.21 ± 0.98 (0.22–4.0)	**0.0001**^**a**^
Surgical treatment	0	50.8%	**0.0001**^**c**^

*Abbreviations*: *IS* idiopathic scoliosis, values shown as mean ± standard deviation (min-max), ^a^ – U-Mann-Whitney test, ^b^ – t-test, ^c^ – Chi^2^ test

significant *P*-values (<0.05) are in bold

We observed significant differences in rs1017861 polymorphism allele distribution (*P* = 0.0030) as well as genotype distribution for an independent and recessive model (*P* = 0.0055 and *P* = 0.0022, respectively). In the case of the rs4738813 SNP, we observed significant differences in allele distribution (*P* = 0.0104) as well as differences shown in genotype distribution for an independent and recessive model of inheritance (*P* = 0.0221, and *P* = 0.0277, respectively). No significant difference in either allele or genotypes distribution was observed in rs4738824 between IS patients with Cobb angle ≤30°(mild IS) and Cobb **≥**30° (moderate or severe IS) (Table 4).

**Table 4:** Allele and genotype distribution between patients with different severity of IS

Allele/genotype	Cobb angle ≤ 30° *N* = 87	Cobb angle ≥ 35° *N* = 118	Independent model *P*-value	*P*-value	OR (CI 0.95)
*rs1017861*					
G	93.7	83.5		**0.0030**^a^	2.93 (1.46–5.91)
A	6.3	16.5			
GG	88.5	69.5	**0.0055**	0.64^b^	2.24 (0.23–21.95)
GA	10.3	28.0			
AA	1.2	2.5		**0.0022**^c^	3.38 (1.6–7.27)
*rs4738824*					
G	83.3	80.5		0.55^a^	1.21 (0.72–2.02)
A	16.7	19.5			
GG	68.9	63.6	0.65	0.91^b^	1.1 (0.18–6.78)
GA	28.7	33.9			
AA	2.3	2.5		0.51^c^	1.27 (0.71–2.29)
*rs4738813*					
T	63.2	75.4		**0.0104**^**a**^	0.56 (0.36–0.86)
C	36.8	24.6			
TT	40.2	56.8	**0.0221**	0.0506^b^	0.36 (0.14–0.94)
CT	44.8	37.3			
CC	14.9	5.9		**0.0277**^**c**^	0.51 (0.29–0.89)

*Abbreviations*: *IS* idiopathic scoliosis, *OR* odds ratio, *CI* 95% confidence interval, P-value for: ^a^ – alleles, ^b^ – dominant model, ^c^ – recessive model

significant *P*-values (<0.05) are in bold

#### Progression rate and polymorphisms association study

The characteristics of patients with IS divided into three subgroups depending on IS progression rate into NP-IS – non-progressive idiopathic scoliosis, SP-IS – slowly-progressive IS and RP-IS – rapidly-progressive IS are listed in Table 5. Similar to the severity of scoliosis, the patients significantly differed in Cobb’s angle and progression rate (*P* = 0.0001). As expected, the surgical treatment was applied more frequent in patients with rapid progression of IS (84.9%).

**Table 5:** Characteristics of patients with different progression rate of IS

	NP-IS *N* = 80	SP-IS *N* = 78	RP-IS *N* = 53	*P*-value
Age (y)	18.5 ± 1.8 (15.0–24.1)	16.9 ± 2.4 (15–26.2)	16.3 ± 7.1 (12.4–50.1)	0.12^b^
Age of menarche	12.9 ± 1.3 (10.0–15.1)	13.1 ± 1.3 (10.0–16.2)	13.1 ± 1.1 (10.5–16.1)	0.22^a^
Cobb angle (°)	23.9 ± 5.4 (10–30)	38.9 ± 7.9 (30–65)	62.7 ± 15.7 (39–114)	**0.0001**^**a**^
Progression rate (°/month)	0.15 ± 0.21 (0.0–1.0)	0.51 ± 0.23 (0.22–0.93)	1.82 ± 0.96 (1.0–4.0)	**0.0001**^**a**^
Surgical treatment	0	12.8%	84.9%	**0.0001**^**c**^

*Abbreviations*: *IS* idiopathic scoliosis, *NP-IS* nonprogressive idiopathic scoliosis, *SP-IS* slowly progressive idiopathic scoliosis and *RP-IS* rapidly progressive idiopathic scoliosis, values shown as mean ± standard deviation (min-max), ^a^- ANOVA with Bonferroni correction, ^b^ –Kurskal-Wallis test, ^c^ –Chi-square test

significant *P*-values (<0.05) are in bold

We observed a significant difference of the rs1017861 polymorphism occurrence between the NP-IS patients vs. the SP-IS patients and the RP-IS patients. This was observed in allele distribution (*P* = 0.0124) and in genotype distribution for both the independent and recessive models (*P* = 0.0316 and *P* = 0.0065, respectively). Analyzing patients with NP-IS vs. RP-IS, we showed significant differences in allele distribution (*P* = 0.0053) as well as in genotype distribution for an independent and recessive model (0.005 and 0.0029, respectively). In the case of the rs4738813 polymorphism, we observed in patients with NP-IS vs. SP-IS and RP-IS differences in alleles distribution (*P* = 0.0338) and in genotypes distribution for an independent and recessive model (*P* = 0.0028 and *P* = 0.0103, respectively). In the case of patients with NP-IS and RP-IS, significant differences in alleles and genotypes distribution were for independent and dominant model (0.0483 and 0.0273, respectively). In the evaluated groups of patients with idiopathic scoliosis, there were no significant differences in alleles and genotypes distribution in the case of rs4738824 and patients with different curve progression rate (Table 6).

**Table 6:** Alleles and genotypes distribution between patients with different rate of progression of IS

				Independent model		Dominant model		Recessive model	
Allele/genotype	NP-IS *N* = 80	SP-IS *N* = 78	RP-IS *N* = 53	*P*-value^a^	*P*-value NP-IS vs. RP-IS^a^	*P*-value^a^	*P*-value NP-IS vs. RP-IS^b^	*P*-value^a^	*P*-value NP-IS vs. RP-IS^a^
rs1017861									
G	93.1	86.5	81.1	**0.0124**	**0.0053**				
A	6.9	13.5	18.9						
GG	87.5	75.6	64.2	**0.0316**	**0.005**	0.83	1	**0.0065**	**0.0029**
GA	11.3	21.8	33.9						
AA	1.3	2.5	1.9						
rs4738824									
G	83.1	80.8	81.1	0.85	0.80				
AA	16.9	19.2	18.9						
GG	68.8	64.1	64.2	0.96	0.80	0.97	1	0.47	0.72
GA	28.8	33.3	33.9						
AA	2.5	2.6	1.9						
rs4738813									
T	62.5	75.	73.6	**0.0338**	0.08				
C	37.5	25.	26.4						
TT	38.8	62.8	50.9	**0.0028**	**0.0483**	0.06	**0.0273**	**0.0103**	0.23
CT	47.5	24.4	47.2						
CC	13.8	12.8	1.9						

*Abbreviations*: *IS* idiopathic scoliosis, *NP-IS* nonprogressive idiopathic scoliosis, *SP-IS* slowly progressive idiopathic scoliosis and *RP-IS* rapidly progressive idiopathic scoliosis, ^a^- Chi-square test

significant *P*-values (<0.05) are in bold

#### Polymorphisms association with IS onset

As in the case of other patients’ assignment, described above, the patients did not differ in age or age of first menarche. The means values observed for Cobb’s angle and progression rate were significantly higher (in both *P* = 0.0001) in EOIS patients. These patients more often undergoing surgical treatment compared to AIS cases (~ 34% higher surgical rate). The characteristics of patients with IS divided into two subgroups into AIS and EOIS is listed in Table 7. In the evaluated groups of patients with IS divided according to the age of onsets scoliosis into AIS and EOIS, there was no difference in alleles and genotypes distribution in rs4738824, rs1017861, and rs4738813 polymorphisms. None of the polymorphism showed a tendency to be in common with the age of IS onset (Table 8).

**Table 7:** Characteristics of patients with different age of IS onset

	AIS *N* = 152	EOIS *N* = 59	*P*-value
Age (y)	18.5 ± 1.8 (12.3–24.1)	16.3 ± 7.1 (12.4–50.1)	0.62^a^
Age of menarche	12.9 ± 1.3 (10.0–15.1)	13.1 ± 1.1 (10.5–16)	0.34^b^
Cobb angle (°)	35.2 ± 15.6 (10–90)	49.4 ± 19.5 (14–114)	**0.0001**^**a**^
Progression rate (°/month)	0.52 ± 0.75 (0–4.0)	1.15 ± 1 (0–4.0)	**0.0001**^**a**^
Surgical treatment	16.4%	50.8%	**0.0001**^**c**^

*Abbreviations*: *IS* idiopathic scoliosis, ^a^ – U-Mann-Whitney test, ^b^ – t-test, ^c^ – Chi-square test

significant *P*-values (<0.05) are in bold

**Table 8:** Alleles and genotypes distribution between patients with different age of onset IS

Allele/genotype	AIS *N* = 152	EOIS *N* = 59	Independentmodel *P*-value	*P*-value	OR (CI 0.95)
*rs1017861*					
G	84.9	89		0.35^a^	0.69 (0.36–1.34)
A	15.1	11			
GG	71.1	79.7	0.44	0.99^b^	0.86 (0.09–8.41)
GA	27	18.6			
AA	2	1.7		0.27^c^	0.63 (0.30–1.29)
*rs4738824*					
G	78	83.1		0.30^a^	0.72 (0.42–1.25)
A	22	16.9			
GG	57.9	69.5	0.27	0.99^b^	1.29 (0.12–14.54)
GA	40.8	28.8			
AA	1.3	1.7		0.16^c^	0.60 (0.32–1.15)
*rs4738813*					
T	66.8	74.6		0.15^a^	1.46 (0.91–2.07)
C	33.2	25.4			
TT	48.7	54.2	0.58	0.45^b^	0.58 (0.19–1.79)
CT	40.1	39			
CC	11.2	6.8		0.57^c^	0.80 (0.44–1.46)

*Abbreviations*: *IS* idiopathic scoliosis, *OR* odds ratio, *CI* 95% confidence interval, *P*-value for: ^a^ – alleles, ^b^ – dominant model, ^c^ – recessive model

## Discussion

It is assumed that differences in *CHD7* gene sequence may increase susceptibility to IS occurrence or/and increase the appearance of phenotypic symptoms but current literature includes conflicting results [9, 14]. Previous findings indicate that approximately 60% of children with a *CHD7* gene mutation, causing CHARGE, develop scoliosis in early childhood [18]. Although the *CHD7* gene has been described as one of the first genes that may be associated with FIS susceptibility [9], the studies regarding this gene are rare and the results are ambiguous. There are only two original papers available that investigated the association of *CHD7* gene SNP with IS [9, 14].

This present study aimed to determine, in one of the Central European populations (Poland), whether there was a relationship between variations in the *CHD7* gene sequence and IS occurrence in young females, as well as a clinical form of this pathology. We did not investigate familial disease associations and none of the patients were related. The patients were diagnosed with IS by a clinician, and the inclusion criterion was the Cobb angle curvature equal 10° or more. Gao et al. used a Cobb angle of 15° as their inclusion criterion [9]. The authors assumed that this threshold would reduce any false positive results. Similar to our study’s approach, Tilley et al. [14] included patients with a Cobb angle greater than 10° and distinguished four subgroups with values of ≥10°, ≥15°, ≥20°, and ≥ 30°. The Cobb angle value of ≥10° used in classifying IS in this study was selected in accordance to the standard criteria recommended by the Scoliosis Research Society [19]. In both the papers mentioned above, the authors researched the familial associations of IS cases with genetic polymorphisms but they did not do a case-control study of genetic alternations occurrence and IS phenotype. All families (USA-citizens) recruited in those studies were Americans of European ancestry [9, 14].

During participant recruitment for our study, bone maturity assessment was important to estimate the IS course. Qualified patients had to meet the criteria indicating the endpoint of spinal growth at the time of the study, as evidenced by age over 15 years, at least 2 years after the first menstrual period, and Risser test score equal 4 or 5. As the patients’ qualification process lasted for several years, these studies were prospective in design. This categorization was not in doubt in the case of patients who required surgical treatment. In these individuals, the criteria of age and bone maturity were not necessary. Inclusion criteria were chosen on the basis of Scoliosis Research Society guidelines in a way that allowed to explicitly exclude other causes of scoliosis. It also made it possible to describe IS clinical course and enabled homogeneous subgroups identification [20].

Patients subgrouping included age of diagnosis, Cobb angle, and the rate of progression (measured by angle curvature changes). The subgroups were comparable regarding anthropological features but different in the course of the disease.

The basic division used in this work distinguished patients due to the curvature at the bone maturity reaching the point. Patients were divided into two groups based on a Cobb angle of ≤30° and **≥** 35°, which has been well accepted as long-term observation of patient with IS has shown curvatures with a Cobb angle above 30° remain stable throughout life and do not progress [21]*.* The curve value of 30° and below ensures a quality of life comparable to a healthy population [21, 22].

The Scoliosis Research Society suggests further division based on the patient’s age at the time of diagnosis, with groupings to distinguish infantile, juvenile and adolescent scoliosis [23]. This division is of great clinical significance since developmental is still ongoing in earlier ages there is a higher likelihood of greater and more complicated deformities of the spine [24]. Since there were no infantile scoliosis cases among the patients qualified for our study, two subgroups were identified: early-onset and adolescent scoliosis.

Tilley et al. divided the patients into four subgroups accordingly with different Cobb angle values. They did not compare them with each other; however, they calculated allele frequencies and identity-by-descent values [14]. In the Gao et al. study, all patients with Cobb angle ≥15^o^ were validated as one subgroup [9]. This made it possible to assess the association of the SNP of the *CHD7* gene with the susceptibility to FIS; however, it did not allow for the investigation into the influence on the clinical form and the course of the disease in non-related cohorts. In this study, we aimed to estimate the relationship between the *CHD7* gene with both susceptibility and the clinical form of scoliosis.

In the studied group of females with IS, the mean age was 16.7 years, the age of menarche onset was 12.9 years, and the average BMI was 18.7. The mean BMI value of this group is at the cut-off value between underweight and normal body mass. Low body mass values are typical in patients with IS [24].

The occurrence of menarche in patients with IS did not differ from the age of menstruation onset in girls who did not suffer from IS. The average age of the first menarche in the Polish population has been estimated to be 13 years [25]. Grivas et al. postulated that the age of menarche onset might be considered as a reliable prognostic factor for IS and varies in different latitudes. The authors associate the late age of menarche with IS susceptibility [26]. In our previous study conducted on the Polish female population diagnosed with IS, we also observed significant differences in the menarche onset time within subgroups divided by the Cobb’s angle curvature. Among females with low (< 30°), mean (30°–49°) and high (≥50°) Cobb angle, we showed that in the group with the highest angular values the first menstruation occurred the latest [27]. In the presented study, with the division of the studied group into scoliosis with Cobb angle ≤30° and **≥** 30°, the menarche onset was not statistically significant (155 ± 16 months vs. 156 ± 15 months). Probably, when applying subgroup division as proposed by Janusz et al. [27] this tendency could be similar, especially that in both studies the values determined for the whole group were very similar (the average time of menarche in the presented studies was 155 ± 15 months, and in Janusz et al. study 154.8 ± 14.7 months).

In these studies, there were no statistically significant differences between patients with rapidly progressive, slowly progressive, and non-progressive scoliosis, in parameters such as patient’s age, the age of the first menstruation onset, body weight and BMI, which infers that these subgroups were comparable in terms of anthropometric features.

No statistically significant differences were found between the patients with early-onset and adolescent scoliosis in age, the age of menarche, body height and weight, and BMI. EOIS scoliosis patients had higher values of the angle of distortion (49.4 ± 19.5° vs. 35.2 ± 15.5°, *P* = 0.0001). Also, a higher progression rate (1.15 ± 1.0° vs. 0.52 ± 0.75° per month, P = 0.0001), and a larger percentage of operations (50.8% vs. 16.4%) was found in this group. This is most likely due to the fact that EOIS appears when the spine is still immature, at a very young age, and there is an extended period of time until the end of growth remains. Additionally, the rapid height increase during the peri-pubertal stage may also be a contributing factor.

### rs13248429, rs78874766 and rs74797613 polymorphisms

For both patients and the control group, only the wild-type allele of rs13248429, rs78874766 and rs74797613 were present. According to available databases, occurrences of these polymorphisms are rare (3‰ -2%). They were included in the study based on the fact that they were located in DNA regions supposedly associated with IS. In the case of polymorphism rs13248429 located in exon 2 (remaining two are mapped in introns), its clinical function is described as follows: the change of the native T allele to mutant A affects the amino acid sequence of the encoded CHD7 protein by tyrosine to asparagine (p.Tyr310Asn) substitution. In the case of rs78874766 and rs74797613, their significance has not been described. However, considering their intragenic location, a possible function would have a more complex character, probably related to the regulation of gene expression or potentially involved in splicing modification. The incidence of individual alleles of the three polymorphisms in the control group corresponded to data for the Central European population described in online databases. Considering the above results and available data, it can be concluded that in the Central European population the above polymorphisms do not show a relationship with either the susceptibility or the clinical form of scoliosis. It seems to be aimless to expand research into these polymorphisms.

### rs1017861 polymorphism

Previously published studies of the IS association with rs1017861 are ambiguous. Gao et al. analyzed both, the linkage of this polymorphism with IS occurring within family members as well as the relationship of haplotypes. The referred authors in the association study point the presence of rs1017861 in relation to the familial disease inheritance [9]. However, in the Tilley et al. study, these results were not confirmed using Stouffer’ Z-score meta-analysis [14]. In our study, we found the association of this polymorphism with the disease susceptibility. The G allele (OR 2.4; *P* = 0.0001) and GG genotype (*P* = 0.0006) of rs1017861 were significantly more frequent in the study group than in the controls, which may indicate its relationship with the IS occurrence. The results vary between both studies as well as our current analysis, this could be explained by the different cohorts which encompasses many deviating aspects such as case selection (FIS vs. case-control study).

There were no significant differences regarding both alleles and genotypes distributions and the clinical form of scoliosis, based on the time of IS onset (EOIS vs. adolescent scoliosis). However, in the case of subgroups Cobb’s angle curvature depending (≥35° and ≤ 30°), statistically significant differences were found. It was shown that the G allele (OR = 2.93, *P* = 0.0030) and the GG genotype (*P* = 0.0055) were more frequent in patients with scoliosis with a curve angle of ≤30°. In the case of subgroups divided by the progression rate, also significant distributions differences in the alleles and genotypes were revealed – the G allele (*P* = 0.01) and the GG genotype (*P* = 0.03) occurred the most frequent in patients with non-progressive scoliosis, less frequently in patients with slow-progressive scoliosis, and the rarest in patients with rapid-progressive scoliosis. Besides the differences in the progression rate, also alleles and genotypes frequencies were analyzed comparing non-progressive and rapid-progressive scoliosis. An even higher statistical significance level was shown. The above presented results suggest that the presence of the G allele and the GG genotype of rs1017861 incline the occurrence of a mild IS clinical course (low Cobb angle, non-progressive form). No former reports were found comparing the frequency of alleles and genotypes of this polymorphism in subgroups of patients with different clinical forms of scoliosis, which makes these results innovative. The modifying effect on the IS has already been described in the context of single nucleotide polymorphisms of other genes [28, 29], which is why this is a proven phenomenon. However, it has not yet been assessed in the context of the *CHD7* gene.

### rs4738824 polymorphism

The rs4738824 association with IS has been described by Gao et al. in FIS linkage studies [9]. Based on haplotype analysis, the authors narrowed the gene fragment, searching polymorphisms of potentially functional implication. These fragments were related to the homeobox protein cdx1, which is associated with the proper axial system during embryonic development in murine models and humans [9, 30, 31]. The authors highlighted the rs4738824 polymorphism, the G > A transition results in disruption of cdx transcription factors binding, which could affect the expression level of the *CHD7* gene and as a result downstream targets for this gene could be affected [9]. Despite the fact of FIS patients in Gao et al. studies, they showed higher G allele frequency in patients with scoliosis [9]. Unfortunately, this polymorphism was not analyzed in the Tilley et al. study [14].

Despite the previously suggested functional role of rs4738824, we did not show statistically significant differences in alleles and genotypes distributions in the case-control study. Similarly, within clinical subgroups, the analysis of the allele and genotype frequencies of this polymorphism in case-by-case study did not show significant differences.

### rs4738813 polymorphism

The rs4738813 association with IS has been described in FIS linkage studies by Gao et al. and confirmed by Krishnan et al. in replication studies [9, 32]. However, the first authors indicate that the results should be handled carefully because of linkage disequilibrium caused by inter-marker occurrence and missing parental genotype information. The observation in the second work suggested in single-point analyses, as well as in multipoint, was significant in patients with a curvature defined at 20° [32]. On the other hand, Tilley et al. did not support this observation in their work [14]. All the authors cited based their work on FIS cases. We did not show statistically significant differences in alleles and genotypes distributions in the case-control study, however, there was strong evidence in genotype as well as alleles distributions in patients with different progression rate. It has also been supported by the observations in patients with Cobb angle ≤30° versus higher curvature. The difference in allels distribution was statistically significant as well as differences in genotypes distriutions for an independent and recessive models. Within adolescent and early onset IS, the distribution of this polymorphism was equal.

Despite the fact that the evidence for rs478813 polymorphism relationship with IS remains inconclusive, this polymorphism was reported in US Patent as the one associated with susceptibility to IS [33].

## Limitations of the study

To confirm our findings, it would be necessary to validate our results in another cohort as our sample size was a notable limitation. Although, it should be emphasized that even with the lower number of participants in each studied group, the Chi-square test was estimated to have high statistical power (99%) of our analysis. However, the obtained results have to be interpreted with certain limitations in mind. While reliable tools of population genetics start from groups of 100 people, they are not always confirmed by the works of other authors. The best evidence for this is the conflicting results associated with the *CHD7* gene obtained by Gao et al. [9] and by Tilley et al. [14]. We should take into consideration that both authors analyzed linkage between *CHD7* gene polymorphisms and FIS, whereas our research was based on a case-control study consisted of non-related individuals.

It should also be considered that the results presented in this study included only six polymorphisms. The *CHD7* gene length and structure (188 kbp, 42 exons), and the possible SNPs number of clinical significance deposited in databases let to suppose, that genetic alternations investigated by our team are only a small part of the *CHD7* gene related to the etiopathogenesis of idiopathic scoliosis.

In the presented work, the study cases consisted entirely of females. This choice was justified by gender-related differences in scoliosis incidence and development. However, in order to determine *CHD7* gene polymorphisms occurrence to the susceptibility of idiopathic scoliosis, it would certainly be necessary to extend this study to the male cases.

## Conclusions

The polymorphism rs1017861 in *CHD7* gene showed an association with IS susceptibility. Two polymorphisms (rs1017861 and rs4738813) were associated with curve severity and progression rate. None of the evaluated polymorphisms in *CHD7* gene showed any association with the age of IS onset. Further evaluation of *CHD7* gene should be considered as IS modifying factor.

## Supplementary information

**Additional file 1: Figure S1.** Standard D’/LOD display obtained from Haploview software. **Table S1.** Linkage data obtained from Haploview software.

## Data Availability

All data analyzed during this study is included in this published article. The raw data is available at the corresponding author upon request.

## Abbreviations

AIS: Adolescent idiopathic scoliosis
CHD7: Chromodomain helicase DNA binding protein 7
EOIS: Early-onset idiopathic scoliosis
FIS: Familial idiopathic scoliosis
IS: Idiopathic scoliosis
NP-IS: Nonprogressive idiopathic scoliosis
OR: Odds ratio
PCR: Polymerase chain reaction
RFLP: Restriction fragment length polymorphism
RP-IS: Rapidly progressive idiopathic scoliosis
SNP: Single nucleotide polymorphism
SP-IS: Slowly progressive idiopathic scoliosis
