# Supplementary material for: CHD7 gene polymorphisms in female patients with idiopathic scoliosis
Source: BMC Musculoskelet Disord. 2020 Jan 10;21:18. doi: 10.1186/s12891-019-3031-0 (PMC6954548; doi:10.1186/s12891-019-3031-0)
Supplement: Supplementary file 1 — Additional file 1: Figure S1. Standard D’/LOD display obtained from Haploview software. Table S1. Linkage data obtained from Haploview software. [file 12891_2019_3031_MOESM1_ESM.docx]

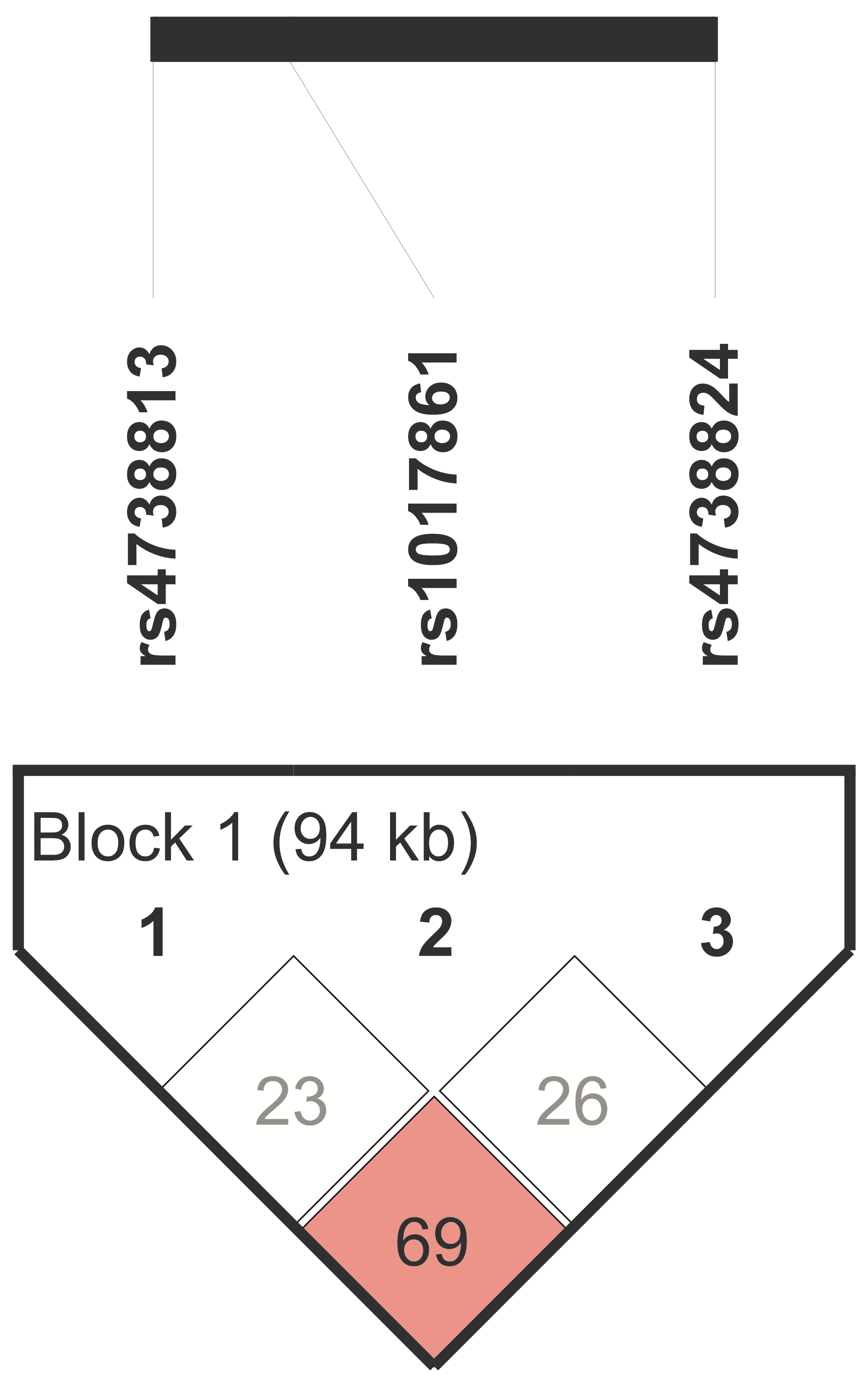


Figure S1. **Standard D’/LOD display obtained from Haploview software.** The values in diamonds show how many of the SNPs in the dataset have been successfully tagged by the set of chosen tests. D’<1 and LOD<2 are indicated in white. D’<1 and LOD≥2 are indicated in pink. There are no D’=1 and LOD≥2. There is strong evidence of recombination, and the highest r^2^ value is 0.284.

Table S1. Linkage data obtained from Haploview software.

| L1 | L2 | D' | LOD | r^2^ | CI low | CI hi |
| --- | --- | --- | --- | --- | --- | --- |
| rs4738813 | rs1017861 | 0.237 | 0.23 | 0.014 | 0.02 | 0.55 |
| rs4738813 | rs4738824 | 0.694 | 8.33 | 0.284 | 0.52 | 0.82 |
| rs1017861 | rs4738824 | 0.26 | 0.59 | 0.028 | 0.04 | 0.5 |

L1, L2 – linkage pairs; D’ – D prime; LOD – logarithm (base 10) of odds, r^2^ – coefficient of determination, CI low/hi – lower and upper confidence r^2^ intervals
